# Risk Factors for Catastrophic Health Events in Head and Neck Cancer: A Scoping Review to Inform Risk Prediction

**DOI:** 10.3390/cancers18122008

**Published:** 2026-06-21

**Authors:** Christabel Oghinan, Deema ElRufaei, Frederick Dun-Dery, Diane Lorenzetti, Sasha Mallya, Andrea S. Fung, Shamir P. Chandarana, T. Wayne Matthews, Tracy Hyndman, Joseph C. Dort, Rui Fu

**Affiliations:** 1 Department of Community Health Sciences, Cumming School of Medicine, University of Calgary, Calgary, AB T2N 4Z6, Canada; christabel.oghinan@ucalgary.ca (C.O.); deema.elrufaei@ucalgary.ca (D.E.); frederick.dundery@ucalgary.ca (F.D.-D.); dllorenz@ucalgary.ca (D.L.); jdort@ucalgary.ca (J.C.D.); 2 Department of Oncology, Cumming School of Medicine, University of Calgary, Calgary, AB T2N 4N2, Canada; sasha.mallya@ahs.ca (S.M.); andrea.fung@albertahealthservices.ca (A.S.F.); shamir.chandarana@ucalgary.ca (S.P.C.); wmatthew@ucalgary.ca (T.W.M.); 3 Department of Surgery, Cumming School of Medicine, University of Calgary, Calgary, AB T2N 2T9, Canada; tracy.hyndman@ucalgary.ca; 4 The Ohlson Research Initiative, Arnie Charbonneau Cancer Institute, Cumming School of Medicine, University of Calgary, Calgary, AB T2N 5G2, Canada

**Keywords:** head and neck cancer, catastrophic health events, adverse events, risk factors, risk prediction

## Abstract

Background/Objectives: Head and neck cancer (HNC) is associated with unique risks of catastrophic health events throughout the care continuum. This scoping review evaluated distinct domains of catastrophic health events in HNC and the associated risk factors to inform the development of risk prediction models. Methods: Five electronic databases were searched to identify peer-reviewed journal articles published from 2015 to 2025. Eligible studies were primary observational studies involving adults (≥18) with HNC that reported a catastrophic (or similar term) health event and assessed the associated risk factors using multivariable regression modelling. Results: Fifty-six studies involving 941,329 patients with HNC were included. Half (*n* = 28, 50.0%) of these studies were conducted in North America, followed by Asia and Europe (*n* = 13 each, 23.2%). Four domains of catastrophic health events were identified: sudden or premature deaths (*n* = 22, 39.3%), severe treatment-related complications (*n* = 19, 33.9%), unplanned acute care encounters (*n* = 22, 39.3%), and severe patient-reported symptoms (*n* = 6, 10.7%). Across all domains, comorbidity was the most repeatedly identified risk factor, followed by treatment characteristics (e.g., surgery duration), older age, and advanced cancer stage. Conclusions: Risk prediction models in HNC should always include variables of comorbidity. The varied measurability of risk factors requires strategic efforts when developing prediction algorithms for different domains of catastrophic health events.

## Simple Summary

Patients with head and neck cancer (HNC) are at risk of experiencing very severe health events, termed catastrophic health events in this paper, during their care journey. We reviewed journal articles published between January 2015 and May 2025 to understand the types of catastrophic health events patients with HNC can experience and risk factors associated with these events. Our review revealed four domains of catastrophic health events for HNC, including sudden or premature deaths, severe treatment-related complications, unplanned acute care use, and severe patient-reported symptoms. Across all domains, comorbidity was the most repeatedly identified risk factor, followed by factors related to the treatment (e.g., surgery duration), older age, and advanced cancer stage. We believe these findings have implications for the development of risk prediction models to help health professionals identify high-risk patients during routine HNC care.

## 1. Introduction

Patients with head and neck cancer (HNC) are at risk of experiencing high-acuity clinical events related to the functionally critical tumour site and complex treatment. These events, termed catastrophic health events in this paper, may occur abruptly and present immediate threats to patient safety and the continuity of cancer care. Catastrophic health events in HNC can either be clinician-reported or patient-reported: for the former, the National Cancer Institute’s Common Terminology Criteria for Adverse Events (CTCAE) provides a standardized framework for clinicians to assess and grade the severity of nearly 900 adverse events [1]; for the latter, which has become increasingly important in cancer research and care delivery [2], patients report symptoms directly using standardized patient-reported outcomes (PRO) instruments such as the PRO-CTCAE [3] and the Edmonton Symptom Assessment System (ESAS) [4].

In HNC, an example of a catastrophic health event is carotid blowout syndrome, which is the sudden rupture of the carotid artery or its branches that results in massive hemorrhage and fatality, if not immediately managed [5]. Although it is relatively rare, carotid blowout has several known risk factors (e.g., prior radiation [6]), offering an opportunity for early detection and intervention. Since some of these risk factors are also associated with other adverse events in HNC (e.g., prior radiation is linked to an increased risk of severe dysphagia [6]), a comprehensive synthesis of these shared risk factors may inform strategies to streamline risk stratification. Several review studies have examined catastrophic health events in HNC, most notably suicide ideation [7,8,9,10,11,12], stage migration [13], treatment-related toxicity [14,15], unplanned acute care encounters [16], and psychological distress [10,17,18,19]. Building on these findings, risk prediction models have been developed for HNC, primarily focusing on survival [20,21,22,23,24] and disease progression [25,26,27,28,29]. Prediction tools that forecast the risk of treatment-related complications [30,31] and unplanned hospital admissions or emergency department (ED) visits [32,33] in HNC are also emerging. There is a gap in the literature regarding (1) a holistic and comprehensive description of catastrophic health events in HNC that are relevant clinically and to patients that should be considered in prediction modelling and (2) insights on common risk factors associated with these catastrophic health events to support the development of such models. As such, our two-fold objective for this scoping review was to (1) characterize catastrophic health events reported for adult patients with HNC and (2) identify risk factors associated with these events to inform risk prediction efforts.

## 2. Materials and Methods

### 2.1. Study Design

This scoping review was reported in accordance with the Preferred Reporting Items for Systematic Reviews and Meta-Analysis extension for Scoping Reviews (Supplementary S1) guidelines [34] using a protocol registered on the Open Science Framework. Following the analytical frameworks by Arksey and O’Malley [35] and Levac et al. [36], two review questions were developed: (1) What catastrophic health events have been reported by adult patients with HNC, and (2) what risk factors have been statistically determined to be associated with these catastrophic health events?

### 2.2. Search Strategy and Eligibility Criteria

Literature search strategies (Supplementary S2), combining controlled vocabulary and keywords, were developed with a medical librarian for the MEDLINE, Embase, CINAHL Plus, APA PsycINFO, and the Cochrane Central Register of Controlled Trials databases, targeting English-language peer-reviewed journal articles published between January 2015 and May 2025. Primary observational studies involving adult (≥18 years) patients diagnosed with HNC that reported a catastrophic health event (see below) and assessed the risk factors using a multivariable regression model were included. Studies that only reported textbook outcomes (e.g., overall survival) or events graded by the CTCAE [1] were excluded to ensure we captured catastrophic health events that have yet to be standardized in reporting. Randomized clinical trials, imaging/tissue analyses, qualitative/mixed-methods studies, and those involving pediatric patients, patients with non-HNC, or had fewer than 11 patients were also excluded.

### 2.3. Study Selection

All citations were uploaded to Covidence. Two reviewers underwent training for title/abstract screen using 100 studies until an 80% agreement was achieved. They then independently screened all titles and abstracts while resolving disagreements with a third reviewer. Studies that passed the title/abstract screen by both reviewers were entered into full-text assessment. Three reviewers underwent training for full-text assessment on a test sample of 10 studies. Working in pairs, they then proceeded with the full-text screen. Studies that passed at least one reviewer were discussed during the weekly research meeting with a fourth reviewer to determine eligibility for inclusion [37].

### 2.4. Study Outcomes

The primary outcome was health-related events for patients with HNC that the study authors termed catastrophic or a related term (such as critical illness; we obtained the list of synonyms for catastrophic from the Thesaurus by Merriam-Webster [38] and confirmed with a medical librarian, see Supplementary S2). Except for mortality, these events were limited to those that occurred within 30 days of the last clinical contact to align with the time window (7–30 days [33,39]) considered to be clinically reasonable for early detection and intervention. The timeframe for mortality was extended to 6 months as deaths represent the most catastrophic event. The secondary outcome was risk factors that were associated with the incidence of a catastrophic health event in a multivariable regression analysis using a 2-sided *p*-value < 0.05 to indicate statistical significance.

### 2.5. Data Extraction, Charting and Synthesis

Four reviewers extracted data using a process that employed the Google NotebookLM (Gemini 3.5), an artificial intelligence (AI)-enabled research tool. First, one reviewer designed two Excel data extraction forms (see data dictionary in Supplementary S3) and used 4 randomly selected studies to conduct a pilot test. For the first form, we extracted the bibliographic information, study design, and the study cohort. For the second form, we extracted the catastrophic health event(s), incidence, and risk factors. The pilot test did not result in any changes to these forms. Next, we provided the pilot-tested results to NotebookLM as prompts to extract data from 10 studies (Supplementary S4), while two reviewers manually and independently extracted data from the same studies. Results were compared during a research meeting with two other reviewers to identify inconsistencies. These insights led to refined prompts. For the remaining studies, we first used NotebookLM to extract data, followed by manual checking by one reviewer. Disagreements were resolved through discussion.

To facilitate data synthesis, three reviewers classified the identified catastrophic health events into four domains following an iterative process: first, based on the extracted definition of the catastrophic health event from each study, reviewers independently formulated a set of domains using common terminologies from the literature [7,8,9,10,11,12,14,15,16,17,18]. Notably, PROs were gathered into a single domain to highlight the unique nature of patient-reported events when compared to clinician-identified events [2]. Then, through discussion, reviewers collapsed these domains into four mutually exclusive ones, including (1) sudden or premature deaths, (2) severe treatment-related complications, (3) unplanned acute care encounters, and (4) severe patient-reported symptoms. For each domain, we mapped risk factors into common categories (sex, age, comorbidity, socioeconomic status, HNC site, advanced cancer stage, and treatment) and further stratified them by the timing of measurement. For comorbidity, we distinguished variables that represented a standard composite index and specific health conditions that may be especially meaningful for the HNC patient population. A narrative approach was used to present these findings.

## 3. Results

### 3.1. Study Inclusion

**Figure 1:**
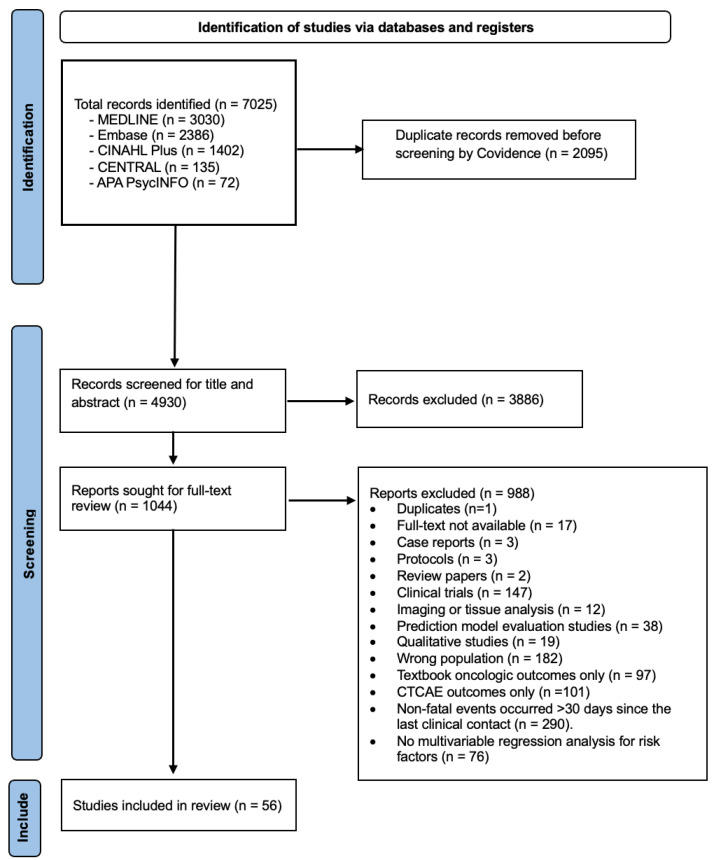
PRISMA flow diagram presenting the inclusion of studies for this review. Abbreviation: CENTRAL, Cochrane Central Register of Controlled Trials; CINAHL, Cumulative Index to Nursing and Allied Health Literature; APA, American Psychological Association; CTCAE, Common Terminology Criteria for Adverse Events.

The search strategy yielded 7025 citations, of which 4930 were unique. After title and abstract screening, 1044 articles were selected for full-text screening. A total of 56 studies involving 941,329 patients with HNC were ultimately included (Figure 1).

### 3.2. Study Characteristics

In Table 1, we show that half (*n* = 28) of the 56 studies were conducted in North America (including 27 from the US [23,40,41,42,43,44,45,46,47,48,49,50,51,52,53,54,55,56,57,58,59,60,61,62,63,64,65] and one from Canada [39]), followed by Asia (*n* = 13, 23.2%, including six from Taiwan [66,67,68,69,70,71], four from China [30,72,73,74], two from South Korea [75,76], and one from Japan [77]), Europe (*n* = 13, 23.2%, including one each from Italy [78], Spain [79], Norway [80], Switzerland [81], and the UK [82], three each from Germany [31,83,84] and Sweden [85,86,87], and two from Finland [88,89]), and Oceania (*n* = 2, 3.6%, one from Australia [90] and the other using a cohort containing patients from both Australia and New Zealand [91]). For study design, most (*n* = 48, 85.7%) used a retrospective cohort, including 15 (26.8%) that used a single-centre cohort [23,31,46,54,55,63,64,69,72,73,75,76,81,84,88], eleven (19.6%) that used a multi-centre cohort [30,41,42,57,70,71,77,78,83,90,91], and 22 (39.3%) that used a provincial, national or international cohort [39,40,43,44,45,47,48,49,50,51,52,53,56,58,59,61,65,67,68,82,86,89]. Other study designs included cross-sectional (*n* = 2, 3.6%) [60,62] and a prospective cohort (*n* = 6, 10.7%) [66,74,79,80,85,87].

Across these studies, sample size ranged from 63 to 275,195. The mean age of patients ranged from 50 to 85 years, and the proportion of men ranged from 38.4% to 94.2%. For cancer site, while eight (14.3%) assessed HNC overall [23,43,49,51,55,56,68,91], the remaining studies focused on specific subsites, most notably the oropharynx (*n* = 9, 16.1%) [40,41,57,58,80,83,85,86,90], the oral cavity (*n* = 8, 14.3%) [46,67,69,73,76,82,84,89], and the larynx (*n* = 6, 10.7%) [42,45,47,48,78,79]. Advanced disease (pathologic or clinical stage III or IV) occurred in 12.5% to 100.0% of patients. Most (*n* = 48, 85.7%) studies included only patients undergoing curative treatment [23,30,31,40,41,42,43,44,45,46,47,48,49,50,51,52,54,55,56,57,58,59,60,61,62,63,64,65,66,67,68,69,72,73,74,75,76,77,78,79,81,82,84,85,87,89,90,91].

### 3.3. Catastrophic Health Events

In Table 2, we summarize catastrophic health events reported by the 56 studies using one row for each reported event.

Sudden or premature deaths were reported by 22 (39.3%) studies [23,40,41,42,50,51,52,60,61,62,65,67,68,70,71,82,83,85,86,88,89,91]. Three studies [52,83,85] reported the incidence of death after receiving a new diagnosis of HNC to be 1.8% within the first 30 days [83] and 3.7% for the first 6 months [85]. Suicide occurred in 1.4% of patients following a diagnosis of laryngeal cancer [52]. Twelve studies reported deaths within 30 days after a HNC surgery [40,41,42,50,51,60,61,62,67,82,88,91], including four studies focusing on in-hospital deaths [60,67,82,91] and one that examined cardiovascular-related deaths [88]. Incidence of deaths in these studies was between 0.25% and 3.4%. One study documenting deaths in the first 90 days of surgery reported an incidence of 10.4% [23]. Three studies focused on deaths during or shortly after the completion of non-surgical treatment [68,86,89] and reported the incidence to range from 2.2% to 10.7%. Three studies [65,70,71] observed deaths within 30 days of a hospital or ED discharge and reported the incidence to be between 1.5% and 15.0%.

Severe treatment-related complications were examined by 19 (33.9%) studies [23,30,31,41,48,51,60,62,67,70,71,72,74,76,77,78,81,82,88]. For carotid blowout and hemorrhage, three studies reported an incidence of 2.2–2.6% within 30 days of surgery [41,73] and up to 23.0% for a rebleeding event among those previously presented to an ED with severe bleeding [70]. For other postoperative complications, fourteen studies found 1.5–43.3% of patients experienced a major complication within the first 30 days [23,30,31,48,51,60,62,66,67,72,78,81,82,88]. For non-surgical treatment, a study of patients undergoing chemotherapy [77] reported that 18.6% and 38.8% of them experienced severe infections and febrile neutropenia, respectively, while a study of patients receiving radiotherapy reported 39.0%, 39.0%, and 20.3% of them developed leukopenia, bone-marrow toxicity, and gastrointestinal injury, respectively [74].

Unplanned acute care encounters were documented by 22 (39.3%) studies. All but one reported these encounters after a HNC surgery. Within the first 30 days after surgery, eighteen studies reported the incidence of an unplanned hospital readmission to be 2.1–58.8% [41,42,45,46,47,49,50,54,55,56,57,58,59,63,65,69,79,90], five studies reported the incidence of an unplanned reoperation was 5.8–20.0% [41,43,44,47,75], and four studies reported the incidence of ED visits to be 5.2–17.5% [54,55,69,79]. In one study reporting unplanned ED visits or hospital admissions within 14 days after an outpatient oncologic visit involving an ESAS assessment [39], the incidence of an ED visit without hospitalization was 5.5% and that of an unplanned hospital admission was 1.6% while a total of 27.0% of patients had at least one such encounter.

Severe patient-reported symptoms were examined in six (10.7%) studies, including three that focused on patients undergoing radiotherapy [66,80,87], two that assessed patients recovering from surgery [64,84], and one that examined those hospitalized for HNC [53]. During radiotherapy or shortly (30-day) after completion, 46.2% of patients reported moderate-to-severe dry mouth [66], while 20% and 25% of them reported moderate or severe pain, respectively [80]. One study further reported that 0.8% of patients initiated opioid use to manage pain [87]. During the first week after surgery, 98.2% of patients reported swallowing difficulties [84], and during the first 3 days of surgery, 22.4–27.6% of patients reported severe pain [64]. Suicidal ideation was reported by 0.78% of patients hospitalized for a skull tumor [53].

### 3.4. Risk Factor for Catastrophic Health Events

The risk factors for each domain of catastrophic health events are presented in Table 2. For sudden or premature deaths (*n* = 22), the literature found older age (*n* = 11) and comorbidity (*n* = 14) were the most common risk factors (Table S1). Upon receiving a HNC diagnosis, older age was reported by all three studies to be a risk factor for death [52,83,85] with older men being a particularly high-risk group [83,85]. For deaths that occurred 30 to 90 days postoperatively, older age was a risk factor in 7 of the 13 studies [23,40,41,60,61,88,91]. Two of the three studies documenting deaths during non-surgical treatment also identified older age as a risk factor [68,89]. For comorbidity, having specific conditions including diabetes [51], chronic obstructive pulmonary disease [41,68], sarcopenia [67], coronary artery disease [88], dysphagia [23], frailty [92], bleeding disorder [41], weight loss before surgery [42], a lower fat-free max index at diagnosis [86], and an overall high burden on the Charlson Comorbidity Index (CCI) [61], the Adult Comorbidity Evaluation-27 system [23], or the Acute Physiology, Age, and Chronic Health Evaluation III (at time of ICU admission [91]) were associated with a higher risk of death. Two studies also reported tobacco use [41] and alcohol consumption [82] as risk factors for death.

Other risk factors for sudden or premature deaths were patient sex (*n* = 2), socioeconomic status (*n* = 3), HNC site (*n* = 4), advanced cancer stage (*n* = 8), and treatment (*n* = 10; Table S1). Patients who were male [83,85], White [52], experienced socioeconomic deprivation [82] or were not covered by any private insurance [60] were at a higher risk of sudden or premature death. Those with tumours of the hypopharynx had a 2.5-fold (vs. the oropharynx [85]) and 3.3-fold (vs. the lip [83]) higher risk of death within 6 months of diagnosis, while those with supraglottic tumour had the highest risk of suicide [52]. Having an advanced-stage tumour conferred strong risk for death [40,52,61,70,82,83,85,89]. Within surgically treated patients, pre/intraoperative risk factors for sudden or premature deaths after the surgery included having an emergency surgery [60,82] or a highly invasive surgery such as a free flap reconstruction [23,60,82], using a 2-team approach [62], and using mechanical ventilation during surgery [91]. Postoperative risk factors included having an extended hospital length of stay [42], an unplanned hospital admission in the first 30 days [42,50] or experience specific complications especially a cardiac event and acute renal failure [51]. For non-surgically managed patients, having received no cancer-directed treatment [83], treatment nonresponse [86], or a history of radiation [52] were risk factors for death. Type of institutions, particularly non-teaching or low-volume hospitals, was also a risk factor [61,65,92].

For severe treatment-related complications (*n* = 19), the most common risk factors were comorbidity (*n* = 15) and treatment factors (*n* = 13; Table S2). For hemorrhage and carotid blowout, risk factors were diabetes [41], underweight [70], or an American Society of Anesthesiologists (ASA) Physical Status Classification III or above [41]. For other complications, diabetes [51], cigarette or alcohol use [82], frailty [30,60], chronic obstructive pulmonary disease [48], coronary artery disease [88], sarcopenia [67], hepatitis [74], tube feeding [77,81,82], underweight [30], and a high overall burden according to the ASA Classification [23] or CCI [31,77] were risk factors. In terms of the treatment, risk factors for hemorrhage or carotid blowout were prior chemoradiation [70] and surgical site infections and flap necrosis developed after surgery [71]. Within surgically treated patients, pre/intraoperative risk factors for postoperative complications were prior radiation or chemotherapy [81], having an emergency admission [82,92], having a highly invasive surgery [31,48,60,78,81,82], using a 2-team approach [62], and a long surgery duration [23,62,78]. Postoperative risk factors for complications included using high-dose glucocorticoid [72] and severe hypoalbuminemia [76]. Receiving the Taxotere-platinol-fluorouracil regime [77] or intensity-modulated radiation therapy [74] was associated with toxicity and injury events in patients undergoing radiation and/or systemic therapy.

In addition to comorbidity and treatment factors, risk factors for severe treatment-related complications included patient sex (*n* = 1), older age (*n* = 2), socioeconomic status (*n* = 1), and HNC site (*n* = 2; Table S2). Women with nasopharyngeal carcinoma had more than 2-folds higher risk of bone-marrow toxicity than their male counterparts [74]. Older age was associated with a higher risk of 30-day postoperative complications [82,88]. Socioeconomic deprivation was associated with higher risks of infections, tracheostomy/gastrostomy malfunction, and complications of the heart and the respiratory tract after surgery [82]. Having tumours in the base of tongue was associated with a higher risk of postoperative hemorrhage [41], while having a second primary cancer or tumour of the larynx was associated with a higher risk of ED presentation due to severe rebleeding [70].

For unplanned acute care encounters (*n* = 22), the most common risk factors were comorbidity (*n* = 17) and treatment (*n* = 15; Table S3). For comorbidity, high-risk surgical patients were those having diabetes [41,49,57], liver disease [45], heart disease [41,42,45,57], depression or other psychiatric conditions [53,54,59], frailty [65], malnutrition [69,79], dysphagia [54], or were a smoker [41,47] before surgery, as well as those scoring high on the CCI [42,50,58,69] or the ASA [41,56,79] at surgery. Preoperative risk factors for a readmission, reoperation or an ED visit 30 days after surgery were prior chemotherapy or radiation [58,75,90], steroid use [47], tracheostomy use [42], and having received a delayed gastrostomy tube placement [55]. With respect to the surgery, having a major surgery, especially flap procedures [42,45,56,57,58,69], a procedure requiring an extended surgery duration [44,75], and admission through the ED [58] were risk factors. Outcomes of the surgery that were risk factors for a subsequent unplanned acute care use were wound contamination or disruption [43,44,46,47,49,56], surgical site infections [43,44,46,49,56], sepsis [49], requiring blood transfusion within 72 h [43,49,56] or ventilator dependence of 48 h [43,44,45], having developed severe acute complications including pneumonia, a cardiovascular event, and dysphagia [42], as well as having a prolonged postoperative length of stay [42,45,58,79].

Other risk factors for unplanned acute care encounters were patient sex (*n* = 2), older age (*n* = 3), socioeconomic status (*n* = 8), HNC site (*n* = 2), advanced cancer stage (*n* = 13), and patient-reported symptoms (*n* = 1; Table S3). Men were nine times more likely to have a 30-day unplanned postoperative readmission than women [54] and around 13% more likely to have a 14-day unplanned ED visit or a hospital admission following an outpatient cancer visit [39]. Older age was also associated with a higher risk [42,65,69], as well as being divorced/separated [42], identifying as Black [44], having no private insurance [45,50,58,63], or being a non-major urban or rural resident [39]. One study found that patients who were relatively affluent had a higher risk of unplanned readmissions [90]. Patients with tumours of a non-buccal site [69] especially the hypopharynx, oropharynx, pharynx, and the larynx [57] were high-risk populations. Advanced cancer stage was a strong risk factor in 13 studies [42,43,44,46,49,50,56,57,58,63,69,75,90]. One study reported high ESAS scores, including individual symptoms or an overall high burden, to be a strong predictor of 14-day unplanned ED visits or hospital admission [39].

For severe patient-reported symptoms (*n* = 7), the most common risk factors were the treatment (*n* = 5), advanced cancer stage (*n* = 3), and comorbidity (*n* = 3; Table S4). For patients undergoing radiation, risk factors of dry mouth or pain were having a high dose of radiation [66], concurrent chemotherapy [87], and having had no surgery prior to radiation [80]. For surgical patients, having a flap reconstruction [84] was a risk factor for impaired swallowing. Having an emergent admission and a long hospital length of stay were risk factors for suicide ideation [53]. An advanced cancer stage was a risk factor for severe symptoms [64,84,87]. Comorbidities, including psychiatric conditions and substance abuse [53], as well as baseline symptoms of dry mouth, difficulty swallowing, mouth sores, sleep disturbance, and fatigue [80,84,87], were all risk factors. Other risk factors were younger age at hospital admission (*n* = 2) [53,64], low social support or socioeconomic status (*n* = 2) [53,80] and pharyngeal cancer (*n* = 1) [87].

## 4. Discussion

In this scoping review, comorbidity was the most repeatedly identified risk factor for catastrophic health events in HNC across four distinct domains. Notably, scoring high on the generic comorbidity scale (including the ASA and CCI) or having specific health conditions, especially diabetes, coronary artery disease, chronic obstructive pulmonary disease, sarcopenia, being underweight, and psychiatric conditions were consistently found to be some of the most important risk factors for catastrophic health events in HNC. Other common risk factors included treatment-level characteristics (for severe treatment-related complications, unplanned acute care use, and severe patient-reported symptoms), older age (for sudden or premature deaths), and advanced cancer stage (for severe patient-reported symptoms). These findings have implications to the development of risk prediction and stratification tools that HNC providers can use to proactively identify high-risk patients during routine care.

Expanding on existing reviews [7,8,9,10,11,12], the first domain of catastrophic health events in this review was deaths that occurred unexpectedly after the last clinical contact. Our results indicated that the time shortly after receiving an HNC diagnosis to be a vulnerable period for suicide and other causes of death, especially for older men diagnosed with advanced HNC. Overwhelming evidence confirmed the HNC surgery to be a procedure associated with elevated mortality risk, as 0.25–3.4% of patients died perioperatively or within the first 90 days postoperatively, with comorbidity and highly invasive or emergent surgery further increasing the risk. Non-surgical treatment was assessed less frequently according to this review; still, 2.2–10.7% of patients died within the first 6 months of treatment completion, especially those who were older. After a hospital or ED discharge, up to 15% of patients died within 30 days. These alarming data highlight the need to continuously track the mental health and physical status of patients with HNC as they are at risk of death throughout the care journey. Indeed, the most repeatedly identified risk factors for death (age and comorbidity) may represent relatively accessible variables through patient charts and electronic medical records (EMRs) in hospital and healthcare system settings. This may imply that sudden or premature deaths should be prioritized in risk prediction effort for HNC due to the importance of such outcomes and the feasibility of being able to capture some of the most important prognostic factors. According to a 2023 review of AI algorithms capable of continuously tracking patient status with EMR data [93], such algorithms do not currently exist for HNC. Hence, future researchers may want to explore the use of continual learning to support the prediction of sudden or premature deaths in HNC [94].

When assessing severe treatment-related complications, risk factors were concentrated in comorbidity and treatment characteristics, particularly those related to surgical complexity and immediate patient outcomes following the surgery. These findings suggest that, unlike sudden or premature deaths, predicting severe treatment-related complications in HNC may require greater reliance on operative notes—where many surgical variables are manually documented—and EMR data to support time-sensitive risk predictions [95]. As such, establishing a centralized, standardized EMR that integrate operative reports (including hand-written ones [96]) should be a priority for hospitals and healthcare systems. Recent advancements in AI have enabled novel ways to record and synchronize anesthesiologic, surgical, and environmental data in an operating room [97]. Examination of data accuracy, patient safety, and impact on patient and clinician privacy is crucial before these technologies can be implemented at a large scale.

Consistent with a 2020 review [16], we found a large body of literature documenting that shortly after HNC surgery, patients tend to experience unplanned readmissions, repeat operations, or ED presentations. Only one study examined unplanned acute care use in non-surgically managed patients [39]. For surgically treated patients, the most commonly reported risk factors were comorbidity measured at surgery and high surgical complexity including complications and other downstream outcomes that occur during or shortly after the surgery. Specifically, surgical site infections and wound dehiscence were repeatedly identified to predict a subsequent unplanned acute care use [16]. Similarly, getting a blood transfusion within the first 72 h, ventilator dependence exceeding 48 h, or having a prolonged hospital length of stay were also found to be some of most important factors leading to an unplanned postoperative acute care encounter. These observations may imply that risk stratification for surgical patients need to be performed at time of surgery and then updated shortly after surgery to allow for the inclusion of intra/postoperative variables to improve the accuracy of predicting the risk of subsequent unplanned acute care encounters. In addition, this is the only domain where socioeconomic factors, notably insurance status [45,50,58,63], rurality [39], marital status [42], race [44], and social deprivation [90], were repeatedly identified as risk factors. These results show that to reliably predict the risk of unplanned acute care use in HNC, the prediction algorithm would require a comprehensively linked dataset-containing patient charts, operative notes and reports, as well as self-reported or population-based registry data capturing socioeconomic factors. This potentially resource-intensive and time-consuming data setup may explain the paucity of prediction modelling studies for this domain in HNC [32,33]. Large language models are promising tools that can support the data pooling and coding of socioeconomic factors for this type of research [98].

We identified some data on severe patient-reported symptoms in HNC. Particularly, severe dry mouth [66] and moderate-to-severe pain [80,87] were reported by patients undergoing radiotherapy, while severe pain [64] and swallowing difficulties [84] were common within the first postoperative week. For these events, risk factors were directly related to the ongoing treatment. These results suggest that collection of PROs should anchor at the beginning of a major cancer-directed treatment and be conducted throughout the course of treatment to closely monitor progression over time. Patients who may require surveillance are individuals who are younger or have pre-existing health conditions, especially symptoms of the head and neck [66], psychiatric conditions [53,80], and substance use [53]. We also note that this is the only domain of catastrophic health events where non-surgically managed patients, especially those undergoing radiotherapy, were somewhat highlighted. Recent advancements in radiotherapy have contributed to better organ-at-risk sparing, toxicity reduction, and improved PROs in HNC. Emerging reviews have documented the efficacy and quality of life benefits of proton therapy [99,100], volumetric modulated arc therapy [101], and an accelerated or hypofractionation therapeutic approach [102,103] for the HNC patient population.

The findings of this review need to be interpreted in the context of the study design and the associated limitations. We did not start the search from database inception; this decision was intentional to capture HNC literature from the past decade to align with paradigm shifts in HNC management, particularly the introduction of immune checkpoint inhibitors [104]. We did not examine non-English articles, despite HNC being the most prevalent in non-English speaking regions of the world [105]. Future studies can overcome this potential language bias by expanding the search strategy. Our literature search yielded a high number of papers focusing on surgical management in HNC with only a few studies documenting non-surgically treated patients and catastrophic health events that can occur in this patient population. This is a limitation of this review considering that novel non-surgical technologies are emerging for HNC including immunotherapy and advancements in radiotherapy. On the same note, due to the low number of non-surgical studies, we also did not summarize the risk of catastrophic health events across different treatment modalities. Future researchers can expand our search strategy to intentionally look for non-surgical treatment modalities in HNC (such as radiotherapy) to capture these important studies. We did not synthesize strategies for prevention and management of the identified catastrophic health events in HNC. The purpose of this review was to provide a comprehensive description on catastrophic health events in HNC and the associated risk factors to guide the conceptualization and development of risk prediction models. To identify catastrophic health events, we required the study authors to explicitly label their assessed outcomes as catastrophic or a similar keyword (see Supplementary S2), which may have resulted in us overlooking studies reporting events that did not contain these keywords but were otherwise considered clinically significant (e.g., hearing loss). Future studies can broaden the scope of this review to capture additional events that are important clinically and to patients/families. We set a 30-day window for all non-fatal catastrophic health events, despite some taking months to develop after diagnosis or first treatment (e.g., a vascular fistula). The purpose of this review was to provide findings that can inform the development of risk prediction models to enable early detection of catastrophic health events during routine HNC care; therefore, we intentionally limited events to those that can occur within a clinically predictable and manageable window (7–30 days [33,39]) since the last clinical encounter. We relied solely on results of multivariable regression modelling to identify statistically meaningful risk factors for catastrophic health events without further considering between-study heterogeneity that may have contributed to these results. This requires a systematic review to critically appraise the reporting quality of each study. We also did not assess the measurability, applicability, or quality of the identified risk factors for each catastrophic health event. Future research can expand our scoping review by extracting the data sources used to measure risk factors in each study to comment on data quality and the feasibility of harmonizing data for inter-jurisdictional model development and validation.

## 5. Conclusions

We reviewed 56 studies involving more than 941,000 adult patients with HNC to understand catastrophic health events that can happen during their care journey and the associated risk factors. Our findings suggest four domains of catastrophic health events, with comorbidity being the most commonly reported risk factor, followed by treatment-related variables (including intraoperative and other downstream consequences of treatment especially surgery), older age, and advanced cancer stage. These findings imply that the development of risk prediction algorithms for HNC should always include variables of comorbidity. Due to the importance of capturing patient outcomes during or shortly after surgery for time-sensitive prediction of subsequent complications, unplanned acute care encounters, deaths or other catastrophic health events, future research needs to elucidate the optimal method of building a centralized EMR that includes operative reports and records of intraoperative and postoperative patient outcomes. Advanced AI techniques that can harmonize data in an operating room to allow for continuous risk prediction should be explored. Considering the varied levels of feasibility of measuring these risk factors, strategic efforts to develop algorithms that can enable early patient risk-stratification during routine HNC care are required.

## Figures and Tables

**Table 1:** Study characteristics (*n* = 56).

Source	Country	Study Design	Study Cohort	Age ^1^	Male	Cancer Site	Advanced Disease ^2^	Treatment Intent
Astrup 2015 [80]	Norway	Single-centre pros cohort	Adults undergoing radiation (*n* = 133)	60 ± 11	71.0	Oropharynx	69	Both
Badr 2019 [54]	USA	Single-centre retro cohort	Adults seen for pre-surgical consult for HNC surgery (*n* = 166)	64 ± 11	75.0	Oral cavity, pharynx, larynx, paranasal sinus/nasal cavity	61.0	Curative
Bazina 2025 [89]	Finland	National retro cohort	Adults diagnosed with HNSCC (*n* = 718)	18+	70.0	Oral cavity	42.0	Curative
Bollig 2022 [40]	USA	National retro cohort	Adults treated surgically (*n* = 785)	58.1 ± 15.0	40.0	Oropharynx	13.9	Curative
Carniol 2017 [49]	USA	National retro cohort	Adults undergoing HNC surgery with free flap reconstruction (*n* = 1204)	18+	65.5	Unspecified	Unknown	Curative
Chaudhary 2017 [42]	USA	Multi-centre retro cohort	Older adults (≥66) undergoing primary HNC surgery (*n* = 1518)	Mean 74.4	79.0	Larynx	22.4	Curative
Chiesa-Estomba 2022 [79]	Spain	Single-centre pros cohort	Adults undergoing HNC surgery (*n* = 342)	59 ± 15	68.7	Larynx	Unknown	Curative
Chiou 2024 [69]	Taiwan	Single-centre retro cohort	Adults undergoing oral cancer resection/reconstruction (*n* = 386)	56.9 ± 10.1	93.5	Oral cavity	72.8	Curative
Choi 2018 [75]	South Korea	Single-centre retro cohort	Adults undergoing curative HNC surgery with or without a flap reconstruction (*n* = 574)	58.8 ± 12.4	78.4	Oropharynx, larynx, hypopharynx	36.0	Curative
Chung 2025 [53]	USA	National retro cohort	Adults hospitalized for skull base tumour (*n* = 275,195)	62.9 ± 17.5	38.4	Skull base	Unknown	Both
Crosetti 2016 [78]	Italy	Multi-centre retro cohort	Older adults (≥70) with SCC (*n* = 212)	75.8 ± 4.5	93.9	Larynx	36.3	Curative
Ferrandino 2018 [45]	USA	National retro cohort	Adults undergoing total laryngectomy (*n* = 2931)	18+	73.9	Larynx	Unknown	Curative
Foley 2023 [90]	Australia	Multi-centre retro cohort	Adults diagnosed with HNC in Queensland (*n* = 1991)	18+	77.7	Oropharynx	73.1	Curative
Frauenfelder 2021 [91]	Australia, New Zealand	Multi-centre retro cohort	Adults admitted to an ICU after HNC surgery (*n* = 10,721)	Median 64.1, range 54.4–73.5	71.6	Unspecified	Unknown	Curative
Frenkel 2018 [55]	USA	Single-centre retro cohort	Adults undergoing a transoral robotic surgery (*n* = 441)	55 or older, 70.3%	78.0	Unspecified	Unknown	Curative
Garg 2017 [56]	USA	National retro cohort	Adults undergoing HNC resection and reconstruction (*n* = 1063)	65 or older, 46.8%	62.7	Unspecified	Unknown	Curative
Ghiam 2018 [57]	USA	Multi-centre retro cohort	Adults undergoing HNC surgery (*n* = 18,121)	61.7 ± 11.6	70.4	Oropharynx	Unknown	Curative
Goel 2019 [58]	USA	National retro cohort	Adults undergoing HNC surgery (*n* = 16,902)	60 or older, 53.1%	73.1	Oropharynx	Unknown	Curative
Haapio 2016 [88]	Finland	Single-centre retro cohort	Adults undergoing HNC surgery (*n* = 456)	Mean 62, range 23–93	66.7	Oral cavity, pharynx, larynx, salivary glands, nasal cavity, paranasal sinuses	Unknown	Both
Helman 2017 [47]	USA	National retro cohort	Adults undergoing total laryngectomy (*n* = 871)	63.1 ± 10.9	82.1	Larynx	Unknown	Curative
Klingelhoffer 2019 [84]	Germany	Single-centre retro cohort	Adults with primary oral cancer (*n* = 400)	62.3 ± 11.2	66.3	Oral cavity	28.9	Curative
Koenen 2024 [31]	Germany	Single-centre retro cohort	Adults diagnosed with SCC treated with total laryngectomy (*n* = 148)	64 ± 10	84.0	Larynx, hypopharynx	53	Curative
Kouka 2022 [83]	Germany	Provincial retro cohort	Adults with primary HNC (*n* = 8288)	Median 60	78.9	Oropharynx	55.5	Both
L’Esperance 2018 [23]	USA	Single-centre retro cohort	Older adults (≥80) undergoing ablative HNC surgery (*n* = 219)	Mean 85	60.2	Unspecified	Unknown	Curative
Lee 2015 [66]	Taiwan	Single-centre pros cohort	Adults treated with parotid-sparing helical tomotherapy (*n* = 67)	Mean 46	82.1	Nasopharynx	31.3	Curative
Lee 2015 [76]	South Korea	Single-centre retro cohort	Adults undergoing major surgery for oral SCC (*n* = 337)	Median 57, range 18–84	69.7	Oral cavity	61.7	Curative
Li 2019 [73]	China	Single-centre retro cohort	Adults with oral SCC who had received surgery (*n* = 1513)	60 or older, 41.5%	66.7	Oral cavity	55.1	Curative
Lin 2020 [68]	Taiwan	National retro cohort	Adults with locally advanced HNSCC on chemoradiation (*n* = 16,029)	52.3 ± 10.1	94.2	Unspecified	100	Curative
Luo 2024 [30]	China	Multi-centre retro cohort	Older adults (≥60) undergoing oral cancer resection and free flap reconstruction (*n* = 1197)	60+	N/A	Oral cavity, oropharynx	Unknown	Curative
Luo 2025 [72]	China	Single-centre retro cohort	Older adults (≥60) undergoing HNC surgery with free-flap reconstruction (*n* = 711)	68.5 ± 6.4	64.1	Tongue	38.3	Curative
Madrigal 2023 [59]	USA	National retro cohort	Adults hospitalized for HNC surgery (*n* = 133,018)	Median 63	68.3	Oral cavity, pharynx, larynx	Unknown	Curative
Mirza 2019 [82]	UK	National retro cohort	Adults undergoing first major HNC surgery (*n* = 12,333)	61.8 ± 11.8	71.1	Oral cavity	Unknown	Curative
Nieman 2018 [92]	USA	National cross-sectional	Adults undergoing ablative HNC surgery (*n* = 159,301)	Mean 62	70.0	Oral cavity, larynx, pharynx	Unknown	Curative
Noel 2021 [39]	Canada	Provincial retro cohort	Adults with HNC completed at least one outpatient ESAS assessment (*n* = 11,741)	60 or older, 61.6%	75.4	Oral cavity, oropharynx, larynx, hypopharynx	Unknown	Both
Oliver 2022 [61]	USA	National retro cohort	Adults undergoing surgical and nonsurgical treatment (*n* = 73,661)	Median 60	82.0	Tonsil, base of tongue, oropharynx	31.4	Curative
Osborn 2018 [46]	USA	Single-centre retro cohort	Adults undergoing a flap reconstruction (*n* = 682)	18+	69.4	Oral cavity	Unknown	Curative
Raikundalia 2016 [51]	USA	National retro cohort	Adults undergoing HNC surgical resection (*n* = 31,075)	61.7 ± 12.7	~71.0	Unspecified	Unknown	Curative
Salati 2023 [81]	Switzerland	Single-centre retro cohort	Adults undergoing laryngectomy (*n* = 84)	64.6 ± 9.0	81.0	Laryngo-pharyngeal	Unknown	Curative
Sangal 2018 [44]	USA	National retro cohort	Adults undergoing major HNC surgery (*n* = 1941)	61 or older, 56.9%	66.9	Floor of mouth, larynx, pharynx, mandible	Unknown	Curative
Sasaki 2015 [77]	Japan	Multi-centre retro cohort	Adults receiving chemotherapy (*n* = 129)	Mean 65	85.0	Pharynx, oral cavity	Unknown	Curative
Schaller 2021 [87]	Sweden	Single-centre pros cohort	Adults undergoing radiation (*n* = 63)	Mean 67, range 36–86	62.0	Oral cavity, pharynx, larynx, others	Unknown	Curative
Shaikh 2023 [62]	USA	Single-centre cross-sectional	Adults undergoing a glossectomy and free flap reconstruction (*n* = 839)	60.2 ± 12.4	64.2	Oral tongue	Unknown	Curative
Sindhar 2019 [63]	USA	Single-centre retro cohort	Adults surgically treated for HNSCC (*n* = 657)	62.0 ± 11.3	73.0	Lip, oral cavity, pharynx, larynx	67.0	Curative
Sylvester 2017 [48]	USA	National retro cohort	Adults (≥40) undergoing laryngectomy (*n* = 40,441)	Mean 63	80.3	Larynx	Unknown	Curative
Talani 2024 [85]	Sweden	Multi-centre pros cohort	Adults newly diagnosed with curable HNSCC (*n* = 404)	63 ± 11	71.0	Oropharynx	40.0	Curative
Talani 2024 [86]	Sweden	National retro cohort	Adults undergoing curative treatment (*n* = 16,786)	66.1 ± 12.6	63.8	Oropharynx	49.1	Both
Tu 2023 [52]	USA and China	National (SEER) and a single-centre (China) retro cohort	Adults with laryngeal cancer (*n* = 42,066 SEER; *n* = 4207 Chinese)	18+	~80	Glottis	20.2	Curative
Van Abel 2022 [64]	USA	Single-centre retro cohort	Adults undergoing transoral robotic surgery (*n* = 216)	59.1 ± 8.3	89.4	Base of tongue, tonsil, soft palate, oropharynx	12.5	Curative
Voora 2022 [65]	USA	National retro cohort	Adults undergoing major HNC surgery (*n* = 14,420)	65 or older, 46.1%	70.0	Oral cavity, larynx, pharynx	Unknown	Curative
Wang 2022 [70]	Taiwan	Multi-centre retro cohort	Adults with HNC presenting to the ED with bleeding (*n* = 231)	56.7 ± 10.9	93.1	Oral cavity, pharynx, larynx	68.4	Unknown
Wong 2025 [41]	USA	Multi-centre retro cohort	Adults with SCC undergoing transoral surgery (*n* = 3489)	60.6 ± 10.0	81.5	Oropharynx	Unknown	Curative
Yao 2021 [74]	China	Single-centre pros cohort	Adults with non-metastatic HNC receiving chemoradiation (*n* = 182)	50.2 ± 11.4	80.2	Nasopharynx	69.2	Curative
Yen 2022 [71]	Taiwan	Multi-centre retro cohort	Adults with HNC presenting to an ED with bleeding (*n* = 241)	56.9 ± 11.2	92.5	Oral cavity, pharynx, larynx	68.4	Unknown
Zeng 2023 [67]	Taiwan	National retro cohort	Adults undergoing curative HNC surgery (*n* = 16,293)	56.4 ± 11.2	89.8	Oral cavity	Unknown	Curative
Zhan 2016 [50]	USA	National retro cohort	Adults undergoing surgery for untreated parotid cancer (*n* = 11,394)	18+	48.0	Parotid	27.1	Curative
Zhao 2018 [43]	USA	National retro cohort	Adults undergoing a free flap surgery for HNC (*n* = 1796)	61 or older, 55.2%	64.7	Unspecified	Unknown	Curative

^1^ Patient age was reported as mean ± standard deviation unless otherwise specified. ^2^ We reported the proportion of the study cohort with pathologic or clinical stage III or IV. Abbreviations: HNC, head and neck cancer; SCC, squamous cell carcinoma; ED, emergency department; ICU, intensive care unit; HNSCC, head and neck squamous cell cancer; retro, retrospective; pros, prospective; SEER, Surveillance, Epidemiology, and End Results Programme.

**Table 2:** Catastrophic health events and risk factors (*n* = 56).

Source	Phase of Care	Outcome ^1^	Incidence	Risk Factors
Domain 1—Sudden or premature deaths				
Bazina 2025 [89]	Curative treatment	Early death (6 m after completion)	10.7%	Older age, T4, N2/N3
Bollig 2021 [40]	Post-op	Death	0.25%	Older age, high-grade tumour, T4a, N1
Chaudhary 2017 [42]	Post-op	Death	1.5%	Preop weight loss, hospital LOS ≥ 6 d, 30 d unplanned readmission
Frauenfelder 2021 [91]	Post-op	In-hospital death	ICU 0.7%, hospital 2.7%	Older age, mechanical ventilation, higher APACHE III score
Haapio 2016 [88]	Post-op	Cardiovascular death	1.3%	Older age, coronary artery disease
Kouka 2022 [83]	Post-diagnosis	Death (30 d–180 d)	30 d 1.8%, 90 d 5.1%, 180 d 9.6%	Male, older age, higher T, M1, advanced stage; oral cavity, oropharynx, or hypopharynx tumour (180 d only), no treatment
L’Esperance 2018 [23]	Post-op	Death (90 d)	10.4%	Older age (90–96), severe comorbidity (ACE-27 = 3), dysphagia, large resection
Lin 2020 [68]	Post-treatment	Death (90 d)	6.7%	Older age, coronary artery disease, COPD, cerebrovascular accident, myocardial infarction, peptic ulcer disease, peripheral vascular disease, metastatic solid cancers
Mirza 2019 [82]	Post-op	In-hospital death	3.35%	Socially deprived, emergency surgery, alcohol use, hypopharynx-larynx tumour, trach, highly invasive resection
Nieman 2018 [92]	Post-op	In-hospital death	0.9%	Older age (>80), Medicare/Medicaid, major procedure, pedicled or free flap reconstruction, urgent admission, frailty, low-volume hospital
Oliver 2022 [61]	Post-op	Early death	1.0%	Older age, CCI > 0, higher T, low-volume hospital
Raikundalia 2016 [51]	Post-op	Death	2.1%	Diabetes, cardiac events, acute renal failure
Shaikh 2023 [62]	Post-op	Death	0.5%	Long surgery duration, 2-team approach
Talani 2024 [85]	Post-treatment	Early death	6 m 2.2%, 12 m 6.2%	Lower fat-free mass index at diagnosis, treatment nonresponse
Talani 2024 [86]	Post-diagnosis	Death (6 m)	3.7%	Older age, male, higher WHO Performance Score, hypopharyngeal tumour, advanced TNM
Tu 2023 [52]	Post-diagnosis	Suicide	1.4%	Older age (≥60), White, subglottic or supraglottic tumour, grade III or IV, distant metastasis, SCC, prior radiation
Voora 2022 [65]	Post-discharge	Death	1.47%	Non-teaching hospitals
Wang 2022 [70]	Post-ED visit	Death	15%	Local recurrence
Wong 2025 [41]	Post-op	Death	0.7%	Smoking (within 1 y), severe COPD, bleeding disorder
Yen 2022 [71]	Post-ED visit	Death	8.3%	Heart rate > 100 bpm, requirement for inotropic support
Zeng 2022 [67]	Post-op	In-hospital death	0.58%	Sarcopenia
Zhan 2016 [50]	Post-op	Death	0.3%	Unplanned 30 d readmission
Domain 2—Severe treatment-related complications				
Li 2019 [73]	Post-op	Hematoma, jugular rupture, carotid blowout syndrome	2.2%	Flap necrosis, SSI
Wang 2022 [70]	After ED visit	Rebleeding	23%	Heart rate > 110 bpm, chemoradiation, second primary or laryngeal tumour, underweight
Wong 2025 [41]	Post-op	Hemorrhage	2.6%	Diabetes, ASA class III or above, base of tongue tumour
Crosetti 2016 [78]	Post-op	Complications	20.3%	More invasive open neck surgery, surgery duration > 2 h
Haapio 2016 [88]	Post-op	Major cardiac and cerebrovascular events	7.2%	Older age, coronary artery disease
Koenen 2024 [31]	Post-op	Complications	Grade III–IV 28%, Grade IV 14.2%, death 0.7%	Higher CCI, intraoperative reconstruction
L’Esperance 2018 [23]	Post-op	Serious complications (death, unplanned transfer to ICU or return to OR, readmission < 30 d)	30.0%	ASA class IV or above, surgery duration ≥ 6 h
Lee 2015 [76]	Post-op	Surgical site infection	26.1%	Postop severe hypoalbuminemia
Luo 2024 [30]	Post-op	Complications	Pulmonary 16.5%, delirium 8.1%, flap 6.9%, cardiac insufficiency 5.6%, hepatic insufficiency 5.3%	Malnourished and/or frail (pulmonary or flap complication, acute renal injury, hepatic or cardiac insufficiency, postop delirium)
Luo 2025 [72]	Post-op	Complications	Pulmonary 43.3%, digestive 37.7%, flap 34.5%, atelectasis, 2.1%, hyperglycemia, 55.3%	Using high-dose glucocorticoid after surgery (any complications, atelectasis, hyperglycemia, flap complications)
Mirza 2019 [82]	Post-op	Complications	37.9%	Older age, social deprivation, smoking, alcohol use, emergency surgery, neck dissection, trach
Nieman 2018 [92]	Post-op	Complications	35.6%	Medicaid, frailty, major procedure, pedicled or free flap reconstruction, urgent/emergent admission
Raikundalia 2016 [51]	Post-op	Complications	Cardiac event 4.9%, acute renal failure 1.7%, pulmonary edema or failure, 5.8%	Diabetes (infections, cardiac event, acute renal failure)
Salati 2023 [81]	Post-op	Complications (fistula)	28.6%	Flap reconstruction; prior locoregional radiation, chemo, or trach
Sasaki 2015 [77]	During chemotherapy	Severe infections, febrile neutropenia	Severe infection: 18.6%, febrile neutropenia 38.8%	Severe infections: higher CCI, tube feeding; Febrile neutropenia: TPF regimen, tube feeding
Shaikh 2023 [62]	Post-op	Complications	35.4%	Long surgery duration, 2-team approach
Sylvester 2017 [48]	Post-op	Complications	Pulmonary 22.3%, nonpulmonary surgical 14.3%, nonpulmonary medical, 12.5%	COPD (for pulmonary and nonpulmonary medical complications after partial laryngectomy or just pulmonary complications after a total laryngectomy)
Yao 2021 [74]	During treatment	Leukopenia, bone-marrow toxicity, and GI injury	Leukopenia 39.0%, bone-marrow toxicity 39.0%, GI injury 20.3%	Leukopenia: IMRT, bone-marrow toxicity: female, hepatitis, GI injury: IMRT
Zeng 2022 [67]	Post-op	Major complications	Pneumonia 9.5%, acute renal failure 1.5%, septicaemia 8.4%	Sarcopenia
Domain 3—Unplanned acute care encounters				
Badr 2019 [54]	Post-op	ED visit, readmission	ED visit 6%, readmission 11%	Readmissions: male, psychiatric history, lower preop MDADI scores; ED visits: lower preop MDADI scores
Carniol 2017 [49]	Post-op	Readmission	9.6%	Diabetes, hyponatremia, leukocytosis, SSI, organ/space SSI, wound disruption, blood transfusion < 72 h, sepsis during surgery
Chaudhary 2017 [42]	Post-op	Readmission	214/1518 (14.1%)	Older age (>80), divorced/separated, stage III/IV, CCI > 1, preop trach, major surgical procedure, hospital LOS ≥ 6 d, postop pneumonia, acute cardiovascular event, postop dysphagia
Chiesa-Estomba 2022 [79]	Post-op	Readmission, ED visit	Readmission 11.4%, ED visit 17.5%	High ASA class, malnutrition, hospital LOS > 7 d
Chiou 2024 [69]	Post-op	ED revisit (≤3 d), readmission	ED revisit 12%, readmission 3%	Older age, non-buccal tumour, CCI > 0, lower BMI, lower hemoglobin, lower albumin, bilateral neck dissection
Choi 2018 [75]	Post-op	Reoperation	10.5%	N2, long surgery duration, previous treatment before surgery
Ferrandino 2018 [45]	Post-op	Readmission	17.5%	Medicaid/Medicare, coagulopathy, liver disease, valvular heart disease, pedicle graft/flap, primary tracheoesophageal fistulization, mechanical ventilation < 96 h, hospital LOS ≤ 10 d
Foley 2023 [90]	Post-op	Readmission	58.8%	Affluent socioeconomic status, higher CCI, stage III/IV disease, (chemo)radiation
Frenkel 2018 [55]	Post-op	ED visit or readmission due to poor oral intake	ED visit 5.2%, readmission 5.0%	Delayed gastrostomy tube placement
Garg 2017 [56]	Post-op	Readmission	8.8%	Deep wound infection, wound dehiscence, ASA class IV, disseminated cancer, laryngopharyngectomy, blood transfusion ≤ 72 h
Ghiam 2018 [57]	Post-op	Readmission	13.8%	Hypopharynx, oropharynx, pharynx, larynx tumour, flap procedures, laryngectomy, electrolyte imbalances, diabetes, depression, metastatic cancer, congestive heart failure.
Goel 2019 [58]	Post-op	Non-elective readmission	10.2%	Medicare/Medicaid, higher CCI, total glossectomy, pharyngotomy, or mandibulectomy, prior radiation, index admission via the ED, hospital LOS ≥ 6 d
Helman 2017 [47]	Post-op	Readmission, reoperation	Readmission 11.9%, reoperation 9.5%	Reoperation: preop steroid use, systemic inflammatory response syndrome, Class III contaminated wound, smoking (within 1 y); Readmission: contaminated or dirty/infected wounds (Class IV)
Madrigal 2023 [59]	Post-op	Readmission	10.1%	Depression diagnosis
Noel 2021 [39]	Outpatient clinic visits during routine care	Hospital admission or an ED visit (14 d)	At least one event 27.0% (patients), ED w/o hospital admission 5.5% (visits), admission 1.6% (visits)	Male, non-major urban or rural dwelling, higher ADG, recent receipt of chemoradiation or surgery + chemoradiation, diagnosed in more recent era (2012–2018), high individual symptom scores (except for nausea, anxiety, or depression) or the highest individual symptom score (h-ESAS) from the last ESAS assessment
Osborn 2018 [46]	Post-op	Readmission	19.8%	SSI, open wounds, use of retained hardware
Sangal 2018 [44]	Post-op	Reoperation	14.2%	Black race, disseminated cancer, long surgery duration, SSI, wound dehiscence, ventilator dependence > 48 h
Sindhar 2019 [63]	Post-op	Readmission	11%	Medicare/Medicaid, advanced TMN stage
Voora 2022 [65]	Post-op	Readmission	11%	Older age (>75), Hospital Frailty Risk Score ≥ 5, electrolyte abnormalities
Wong 2025 [41]	Post-op	Readmission, Reoperation	Readmission 8.9%, reoperation 5.8%	Readmission: smoking (within 1 y), congestive heart failure; reoperation: diabetes, ASA class III or above
Zhan 2016 [50]	Post-op	Readmission	2.1%	Uninsured status, advanced pathologic T, CCI > 0
Zhao 2018 [43]	Post-op	Reoperation	20.0%	SSI, wound disruption, blood transfusion < 72 h, ventilation > 48 h
Domain 4—Severe patient-reported symptoms				
Astrup 2015 [80]	During radiation	Self-reported pain	Moderate 20%, severe 25%	Higher comorbidity (Self-administrated Comorbidity Questionnaire-19), difficulty swallowing, mouth sores, sleep disturbance, fatigue, low social support, low KPS, no surgery before radiation
Chung 2025 [53]	Post-diagnosis	Suicidal ideation	0.78%	Younger age, emergent admission, socioeconomic difficulties; depressive, bipolar, adjustment, anxiety, alcohol use, or cannabis use disorder; longer hospital LOS
Klingelhoffer 2019 [84]	Post-op	Swallowing impairment (7 d)	98.2%	Larger tumour size, higher N stage, trach, insufficient dental status, flap reconstruction
Lee 2015 [66]	Post-radiation	Moderate-to-severe xerostomia	46.2%	High mean dose to ipsilateral submandibular gland, the contralateral submandibular gland, or the oral cavity, baseline xerostomia
Schaller 2021 [87]	During radiation	Opioid use for pain	0.78%	Pharyngeal tumour, concurrent chemo, higher mucositis grade
Van Abel 2022 [64]	Post-op	Severe pain (0 d–3 d)	Postop day zero 32.9%, day one 27.6%, day two 22.4%, day three 9.3%	Younger age, high T stage (only days 1 and 2)

^1^ For each catastrophic health event, the time window for its occurrence was within 30 days of the last clinical contact, unless otherwise stated. Abbreviations: ACE-27, the Adult Comorbidity Evaluation-27 scoring system; ICU, intensive care unit; APACHE, Acute Physiology and Chronic Health Evaluation; ED, emergency department; ESAS, Edmonton Symptom Assessment System; ADG, Aggregated Diagnosis Groups; CCI, Charlson Comorbidity Index; ASA, the American Society of Anesthesiologists; MDADI, MD Anderson Dysphagia Inventory; GI, gastrointestinal; IMRT, intensity-modulated radiation therapy; h, hour; d, day; m, month; y, year; postop, postoperative; preop, preoperative; OR, operation room; KPS, Karnofsky Performance Status; BMI, Body Mass Index; COPD, chronic obstructive pulmonary disease; SSI, surgical site infection; TPF, Taxotere-Platinol-Fluorouracil; LOS, length of stay; SCC, squamous cell carcinoma; trach, tracheostomy.

## Data Availability

The study protocol of this scoping review is publicly available on the Open Science Framework via https://osf.io/xq43b/ accessed on 16 June 2026).

## Supplementary Materials

The following supporting information can be downloaded at: https://www.mdpi.com/article/10.3390/cancers18122008/s1, Supplementary S1: Preferred Reporting Items for Systematic reviews and Meta-Analyses extensions for Scoping Reviews (PRISMA-ScR) checklist; Supplementary S2: literature search strategy and results; Supplementary S3: data dictionary for two data extraction forms; Supplementary S4: process of using the Google NotebookLM to assist data extraction; Table S1: mapping risk factors for sudden or premature deaths; Table S2: mapping risk factors for treatment-related complications; Table S3: mapping risk factors for unplanned acute care encounters; Table S4: mapping risk factors for patient-reported severe symptoms.

## Abbreviations

The following abbreviations are used in this manuscript:

ACE-27	Adult Comorbidity Evaluation-27 scoring system
ADG	Aggregated Diagnosis Groups
APA	American Psychological Association
APACHE	Acute Physiology and Chronic Health Evaluation
ASA	American Society of Anesthesiologists
BMI	Body Mass Index
CCI	Charlson Comorbidity Index
CENTRAL	Cochrane Central Register of Controlled Trials
Chemo	Chemotherapy
CINAHL	Cumulative Index to Nursing and Allied Health Literature
COPD	Chronic obstructive pulmonary disease
CTCAE	Common Terminology Criteria for Adverse Events
ED	Emergency department
ESAS	Edmonton Symptom Assessment System
GI	Gastrointestinal
HNC	Head and neck cancer
HNSCC	Head and neck squamous cell carcinoma
ICU	Intensive care unit
IMRT	Intensity-modulated radiation therapy
KPS	Karnofsky Performance Status
LOS	length of stay
MDADI	MD Anderson Dysphagia Inventory
OR	Operating room
PRISMA-ScR	Preferred Reporting Items for Systematic reviews and Meta-Analyses extensions for Scoping Reviews
PRO	Patient-reported outcomes
SCC	Squamous cell carcinoma
SEER	Surveillance, Epidemiology, and End Results
SES	Socioeconomic status
SSI	Surgical site infections
TPF	Taxotere-Platinol-Fluorouracil
