# Supplementary material for: Risk Factors for Catastrophic Health Events in Head and Neck Cancer: A Scoping Review to Inform Risk Prediction
Source: Cancers (Basel). 2026 Jun 21;18(12):2008. doi: 10.3390/cancers18122008 (PMC13296962; doi:10.3390/cancers18122008)
Supplement: Supplementary file 1 [file cancers-18-02008-s001.zip › cancers-4327652-supplementary.pdf]

Risk factors for catastrophic health events in head and neck cancer: a scoping review to inform risk prediction

Supplemental Material

Supplementary S1 Preferred Reporting Items for Systematic reviews and Meta-Analyses extensions for Scoping Reviews (PRISMA-ScR) checklist

Supplementary S2 Literature search strategy and results

Supplementary S3 Data dictionary for two data extraction forms

Supplementary S4 Process of using the Google NotebookLM to assist data extraction

Table S1 Mapping risk factors for sudden or premature deaths

Table S2 Mapping risk factors for treatment-related complications

Table S3 Mapping risk factors for unplanned acute care encounters

Table S4 Mapping risk factors for patient-reported severe symptoms

Supplementary S1 Preferred Reporting Items for Systematic reviews and Meta-Analyses extensions for Scoping Reviews checklist

SECTION	ITEM	PRISMA-ScR CHECKLIST ITEM	REPORTED ON PAGE #
**TITLE**			
Title	1	Identify the report as a scoping review.	1
**ABSTRACT**			
Structured summary	2	Provide a structured summary that includes (as applicable): background, objectives, eligibility criteria, sources of evidence, charting methods, results, and conclusions that relate to the review questions and objectives.	1-2
**INTRODUCTION**			
Rationale	3	Describe the rationale for the review in the context of what is already known. Explain why the review questions/objectives lend themselves to a scoping review approach.	2
Objectives	4	Provide an explicit statement of the questions and objectives being addressed with reference to their key elements (e.g., population or participants, concepts, and context) or other relevant key elements used to conceptualize the review questions and/or objectives.	2
**METHODS**			
Protocol and registration	5	Indicate whether a review protocol exists; state if and where it can be accessed (e.g., a Web address); and if available, provide registration information, including the registration number.	2
Eligibility criteria	6	Specify characteristics of the sources of evidence used as eligibility criteria (e.g., years considered, language, and publication status), and provide a rationale.	2-3
Information sources*	7	Describe all information sources in the search (e.g., databases with dates of coverage and contact with authors to identify additional sources), as well as the date the most recent search was executed.	2
Search	8	Present the full electronic search strategy for at least 1 database, including any limits used, such that it could be repeated.	Appendix S2
Selection of sources of evidence†	9	State the process for selecting sources of evidence (i.e., screening and eligibility) included in the scoping review.	3
Data charting process‡	10	Describe the methods of charting data from the included sources of evidence (e.g., calibrated forms or forms that have been tested by the team before their use, and whether data charting was done independently or in duplicate) and any processes for obtaining and confirming data from investigators.	3; Appendix S3
Data items	11	List and define all variables for which data were sought and any assumptions and simplifications made.	3
Critical appraisal of individual sources of evidence§	12	If done, provide a rationale for conducting a critical appraisal of included sources of evidence; describe the methods used and how this information was used in any data synthesis (if appropriate).	N/A
Synthesis of results	13	Describe the methods of handling and summarizing the data that were charted.	3

SECTION	ITEM	PRISMA-ScR CHECKLIST ITEM	REPORTED ON PAGE #
**RESULTS**			
Selection of sources of evidence	14	Give numbers of sources of evidence screened, assessed for eligibility, and included in the review, with reasons for exclusions at each stage, ideally using a flow diagram.	3-4, Figure 1
Characteristics of sources of evidence	15	For each source of evidence, present characteristics for which data were charted and provide the citations.	4-9; Table 1
Critical appraisal within sources of evidence	16	If done, present data on critical appraisal of included sources of evidence (see item 12).	N/A
Results of individual sources of evidence	17	For each included source of evidence, present the relevant data that were charted that relate to the review questions and objectives.	Table 2; Tables S1-S4
Synthesis of results	18	Summarize and/or present the charting results as they relate to the review questions and objectives.	10-18
**DISCUSSION**			
Summary of evidence	19	Summarize the main results (including an overview of concepts, themes, and types of evidence available), link to the review questions and objectives, and consider the relevance to key groups.	18-19
Limitations	20	Discuss the limitations of the scoping review process.	21
Conclusions	21	Provide a general interpretation of the results with respect to the review questions and objectives, as well as potential implications and/or next steps.	20-21
**FUNDING**			
Funding	22	Describe sources of funding for the included sources of evidence, as well as sources of funding for the scoping review. Describe the role of the funders of the scoping review.	Funding statement, pg 22

JBI = Joanna Briggs Institute; PRISMA-ScR = Preferred Reporting Items for Systematic reviews and Meta-Analyses extension for Scoping Reviews.
* Where *sources of evidence* (see second footnote) are compiled from, such as bibliographic databases, social media platforms, and Web sites.
† A more inclusive/heterogeneous term used to account for the different types of evidence or data sources (e.g., quantitative and/or qualitative research, expert opinion, and policy documents) that may be eligible in a scoping review as opposed to only studies. This is not to be confused with *information sources* (see first footnote).
‡ The frameworks by Arksey and O'Malley (6) and Levac and colleagues (7) and the JBI guidance (4, 5) refer to the process of data extraction in a scoping review as data charting.
§ The process of systematically examining research evidence to assess its validity, results, and relevance before using it to inform a decision. This term is used for items 12 and 19 instead of "risk of bias" (which is more applicable to systematic reviews of interventions) to include and acknowledge the various sources of evidence that may be used in a scoping review (e.g., quantitative and/or qualitative research, expert opinion, and policy document).

From: Tricco AC, Lillie E, Zarin W, O'Brien KK, Colquhoun H, Levac D et al. PRISMA Extension for Scoping Reviews (PRISMAScR): Checklist and Explanation. Ann Intern Med. 2018;169:467–473. doi: 10.7326/M18-0850.

Supplementary S2 Literature search strategy and results

MEDLINE

	Search strings	Hits
Concept #1 = Head and neck cancers		
1	"head and neck neoplasms"/	70166
2	exp "squamous cell carcinoma of head and neck"/	14215
3	exp mouth neoplasms/	81117
4	exp otorhinolaryngologic neoplasms/	100323
5	((head or neck or tongue or tonsil or salivary or tonsillar or pharynx or pharyngeal or hypopharynx or hypopharyngeal or sinus or oropharynx or oropharyngeal or glottic or supraglottic or larynx or laryngeal or nasopharynx or nasopharyngeal or palate or mouth or oral or nose or nasal or ear or auricular or auricle or otorhinolaryn* or epiglottic or throat) adj2 (cancer* or neoplasm* or malignan* or tumor* or tumor* or carcinoma* or epidermoid*)).ab,kf,ti.	150287
6	OR [1-5]	280134
Concept #2 = Carotid blowout		
7	*Carotid Artery Diseases/	19539
8	(carotid ADJ3 (hemorrhage OR bleeding OR blowout OR rupture OR stenosis)). ab,kf,ti.	15158
9	OR [7-8]	32685
Concept #3 = Catastrophic health events		
10	Hospital Mortality/ or Mortality/ or Mortality, Premature/ or Self-Injurious Behavior/	118800
11	Catastrophic Illness/ or Critical Illness/ or Clinical Deterioration/	45090
12	((catastroph* or critical or life-threatening or fatal* or emergen* or severe or detrimental or disast* or crisis* or crises* or unplanned or trauma* or sudden or unexpect* or premature or accident* or destruct* or cataclysmic or acute) adj2 (event* or outcome* or condition* or consequence* or disease* or failure* or fail* or accident* or clog* or rupture* or pain* or toxicit* or infection* or complication* or obstruct* or wound* or distress or dysphagia or depression* or depressed* or inflamm* or stent* or bleed* or rebleed* or recurrence* or recur* or visit* or revisit* or admission* or admit* or readmit* or readmission* or reoperat* or experienc* or report* or inciden* or syndrome* or symptom*)).ab,kf,ti.	1211926
13	(sudden* or premature or early or unexpect*) adj2 (die or died or death* or decease* or fatal*). ab,kf,ti.	90822
14	(suicid* or self-harm* or self-abus* or self-injur* or self-mutilat* or self-damag* or self-inflict*). ab,kf,ti.	120745
15	OR [10-14]	1515346
Combining all three concepts		
16	6 AND 9	751
17	6 AND 15	7408
18	16 OR 17	8001

	Search strings	Hits
19	Limit 18 to (English language and yr='2015 – Current')	3551
20	Animals/not humans/	5305836
21	19 not 20	3497
22	Anonymous.au.	821729
23	21 NOT 22	3492
24	("scoping review" or "systematic review" or "rapid review").m_titl.	321397
25	23 NOT 24	3381
26	Limit 25 to "review articles"	347
27	25 NOT 26	3034
28	Limit 27 to "remove preprint records"	3030

EMBASE

	Search strings	Hits
Concept #1 = Head and neck cancers		
1	"head and neck cancer"/	63219
2	"head and neck carcinoma"/ or "head and neck squamous cell carcinoma"/	36570
3	head cancer/	1976
4	mouth cancer/	23720
5	Neck cancer/	2683
6	Nose cancer/	1849
7	Paranasal sinus cancer/	1708
8	Pharynx cancer/	5544
9	Salivary gland cancer/	3012
10	Tongue cancer/	4121
11	Tonsil cancer/	1311
12	((head or neck or tongue or tonsil or salivary or tonsillar or pharynx or pharyngeal or hypopharynx or hypopharyngeal or sinus or oropharynx or oropharyngeal or glottic or supraglottic or larynx or laryngeal or nasopharynx or nasopharyngeal or palate or mouth or oral or nose or nasal or ear or auricular or auricle or otorhinolaryn$ or epiglottic or throat) adj2 (cancer$ or neoplasm$ or malignan$ or tumor$ or tumor$ or carcinoma$ or epidermoid$)).ab,kf,ti.	192464
13	OR [1-12]	245852
Concept #2 = Carotid blowout		
14	carotid artery diseases/	11821
15	(carotid ADJ3 (hemorrhage or bleeding or blowout or rupture or stenosis)).mp.	25237
16	OR [14-15]	35034
Concept #3 = Catastrophic health events		
17	out-of-hospital mortality/ or in-hospital mortality/ or hospital mortality/ or at home mortality/ or premature mortality/	85641
18	sudden death/	46738
19	suicide attempt/ or suicide/ or * automutilation/	115327

	Search strings	Hits
20	*critical illness or *acute disease/ or life threat/ or urgent visit/ or crisis intervention/ or *"accidents and accident related phenomena"/ or *major injury/	38773
21	((catastroph$ or critical or life-threat$ or fatal$ or emergen$ or severe or detrimental or disast$ or crisis$ or crises$ or unpla nned or trauma$ or sudden or unexpect$ or premature or accident$ or destruct$ or cataclysmic) adj2 (event$ or outcome$ or condition$ or consequence$ or failure$ or fail$ or accident$ or clog$ or rupture$ or pain$ or toxicit$ or infection$ or complication$ or obstruct$ or wound$ or distress or dysphagia or depression$ or depressed$ or inflamm$ or stent$ or bleed$ or rebleed$ or recurrence$ or recur$ or visit$ or revisit$ or admission$ or admit$ or readmit$ or readmission$ or reoperat$ or experience$ or report$ or inciden$ or syndrome$ or symptom$)).ab,kf,ti.	1026896
22	(sudden$ or premature or early or unexpect$) adj2 (die or died or death$ or decease$ or fatal$).ab,kf,ti.	133575
23	(suicide$ or self-harm$ or self-abus$ or self-injur$ or self-mutilat$ or self-damag$ or self-inflict$).ab,kf,ti.	127420
24	OR [17-23]	1400933
Combining all three concepts		
25	13 AND 16	825
26	13 AND 24	6580
27	25 OR 26	7241
28	Limit 27 to (English language and humans and "remove preprint records" and yr = "2015 – Current")	4008
29	Limit 28 to conference abstract status	1378
30	28 NOT 29	2630
31	Limit 30 to article in press status	38
32	30 NOT 31	2592
33	Anonymous.au.	855092
34	32 NOT 33	2548
35	"systematic review" OR "rapid review" OR "scoping review".ti.	671339
36	34 NOT 35	2386

CINAHL Plus

	Search strategy	Hits
Concept #1 = Head and neck cancers		
1	(MH "Head and Neck Neoplasms") OR (MH "Squamous Cell Carcinoma of Head and Neck") OR (MH "Tonsillar Neoplasms") OR (MH "Oropharyngeal Neoplasms") OR (MH "Tongue Neoplasms") OR (MH "Pharyngeal Neoplasms") OR (MH "Parotid Neoplasms") OR (MH "Otorhinolaryngologic Neoplasms") OR (MH "Nose Neoplasms") OR (MH "Nasopharyngeal Neoplasms") OR (MH "Mouth Neoplasms") OR (MH "Maxillary Neoplasms") OR (MH "Lip Neoplasms") OR (MH "Laryngeal Neoplasms") OR (MH "Hypopharyngeal Neoplasms") OR (MH "Salivary Gland Neoplasms") OR (MH "Paranasal Sinus Neoplasms")	41971

	Search strategy	Hits
2	XB (((head or neck or tongue or tonsil or salivary or tonsillar or pharynx or pharyngeal or hypopharynx or hypopharyngeal or sinus or oropharynx or oropharyngeal or glottic or supraglottic or larynx or laryngeal or nasopharynx or nasopharyngeal or palate or mouth or oral or nose or nasal or ear or auricular or auricle or otorhinolaryn* or epiglottic or throat) N2 (cancer* or neoplasm* or malignan* or tumor* or tumor* or carcinoma* or epidermoid*)))	44301
3	OR [1-12]	58830
Concept #2 = Carotid blowout		
4	(MH "Carotid Artery Diseases")	3951
5	TX ((carotid and (hemorrhage or bleeding or blowout or rupture or stenosis)))	6468
6	OR [7-8]	9982
Concept #3 = Catastrophic health events		
7	(MH "Death") OR (MH "Death, Sudden") OR (MH "Death, Accidental") OR (MH "Death, Sudden, Cardiac") OR (MH "Sudden Unexpected Death in Epilepsy") OR (MH "Mortality") OR (MH "Hospital Mortality") OR (MH "Fatal Outcome") OR (MH "Critical Illness")	114247
8	(MH "Suicide") OR (MH "Suicide, Attempted") OR (MH "Suicidal Ideation") OR (MH "Self-Injurious Behavior") OR (MH "Injuries, Self-Inflicted")	39277
9	(MH "Emergency Room Visits") OR (MM "Ambulatory Care") OR (MH "Multiple Trauma") OR (MH "Accidental Injuries") OR (MH "Surgical Wound Infection") OR (MH "Emergency Service") OR (MH "Emergency Medical Services") OR (MH "Accidental Falls")	145711
10	XB (((catastroph* or critica* or life-threat* or fatal* or emergen* or severe or detrimental or disast* or crisis* or crises* or unplanned or trauma* or sudden or unexpect* or premature or accident* or destruct* or cataclysm*) N2 (event* or outcome* or condition* or consequence* or disease* or failure* or fail* or accident* or clog* or ruptur* or pain* or toxicit* or infect* or complicat* or obstruct* or wound* or distress or dysphagia or depression* or depressed* or inflamm* or inflamed or stent* or bleed* or rebleed* or recurrent* or recur* or visit* or revisit* or admission* or admit* or readmit* or readmission* or reoperat* or experienc* or report* or inciden* or syndrome* or symptom*)))	199761
11	XB ((sudden* or premature or earl* or unexpect*) N2 (die or died or death* or decease* or fatal*))	20431
12	XB ((suicide* or self-harm* or self-abus* or self-injur* or self-mutilat* or self-damag* or self-inflict*))	40989
13	OR [7-12]	485542
Combining all three concepts		
14	3 AND 6	306
15	3 AND 13	2009
16	14 OR 15	2267
17	Limit 16 to Scholarly (Peer Reviewed) Journals; Publication Dates: 20150101 – 20251231; English-language.	1402

CENTRAL

ID	Search strings	Hits
#1	MeSH descriptor: [Head and Neck Neoplasms] explode all trees	9530
#2	MeSH descriptor: [Squamous Cell Carcinoma of Head and Neck] explode all trees	716
#3	MeSH descriptor: [Mouth Neoplasms] explode all trees	955
#4	MeSH descriptor: [Otorhinolaryngologic Neoplasms] explode all trees	1887
#5	((head or neck or tongue or tonsil or salivary or tonsillar or pharynx or pharyngeal or hypopharynx or hypopharyngeal or sinus or oropharynx or oropharyngeal or glottic or supraglottic or larynx or laryngeal or nasopharynx or nasopharyngeal or palate or mouth or oral or nose or nasal or ear or auricular or auricle or otorhinolaryn* or epiglottic or throat) NEAR/2 (cancer* or neoplasm* or malignan* or tumor* or tumor* or carcinoma* or epidermoid*)):ab,ti,kw	14045
#6	#1 or #2 or #3 or #4 or #5	18018
#7	MeSH descriptor: [Carotid Artery Diseases] explode all trees	1760
#8	(carotid NEAR/3 (hemorrhage or bleeding or blowout or rupture)).mp.	21282
#9	#7 or #8	23029
#10	MeSH descriptor: [Death, Sudden] explode all trees	1219
#11	MeSH descriptor: [Hospital Mortality] explode all trees	1860
#12	MeSH descriptor: [Fatal Outcome] explode all trees	21
#13	MeSH descriptor: [Mortality, Premature] explode all trees	8
#14	MeSH descriptor: [Self-Injurious Behavior] explode all trees	2635
#15	MeSH descriptor: [Catastrophic Illness] explode all trees	6
#16	MeSH descriptor: [Critical Illness] explode all trees	3773
#17	MeSH descriptor: [Acute Disease] this term only	11683
#18	MeSH descriptor: [Clinical Deterioration] explode all trees	53
#19	MeSH descriptor: [Emergencies] explode all trees	1865
#20	((catastroph* or critical or life-threat* or fatal* or emergen* or severe or detrimental or disast* or cris* or unplanned or trauma* or sudden or unexpect* or premature or accident* or destruct* or cataclysmic or acute) NEAR/2 (event* or outcome* or condition* or consequence* or failure* or fail* or accident* or clog* or rupture* or pain* or toxicit* or infection* or complication* or obstruct* or wound* or distress or dysphagia or depression or depressed or inflamm* or inflamed or stent* or bleed* or rebleed* or recurrence* or recur* or visit* or revisit* or admission* or admit* or readmit* or readmission* or reoperat* or experienc* or report* or inciden* or syndrome* or symptom*)).mp	21282
#21	(sudden* or premature or early or unexpect*) NEAR/2 (die or died or death* or decease* or fatal*).mp	27522
#22	(suicid* or self-harm* or self-abus* or self-injur* or self-mutilat* or self-damag* or self-inflict*).mp	31867
#23	OR [#10-#22]	45052
#24	#6 AND #9	199
#25	#6 AND #23	271
#26	#24 or #25 with Publication Year from 2015 to 2025, in Trials	135

APA PsycINFO

ID	Search strings	Hits
Concept #1 = Head and neck cancers		
1	(exp "Neck (Anatomy)"/or exp "Head (Anatomy)"/) AND neoplasms/	406
2	((head or neck or tongue or tonsil or salivary or tonsillar or pharynx or pharyngeal or hypopharynx or hypopharyngeal or sinus or oropharynx or oropharyngeal or glottic or supraglottic or larynx or laryngeal or nasopharynx or nasopharyngeal or palate or mouth or oral or nose or nasal or ear or auricular or auricle or otorhinolaryn* or epiglottic or throat) adj2 (cancer* or neoplasm* or malignan* or tumor* or tumor* or carcinoma* or epidermoid*)).mp.	1960
3	OR [1-2]	1960
Concept #2 = Carotid blowout		
4	Carotid Arteries/	1305
5	(carotid ADJ5 (hemorrhage or bleeding or blowout or rupture or stenosis)).mp.	255
6	OR [4-5]	1475
Concept #3 = Catastrophic health events		
7	Sudden Death/ or Brain Death/ or Self-Destructive Behavior/	4300
8	Emergency Services/ or Cerebrovascular Accidents/ or Acute Illness/ or Acute Pain/ or Acute Schizophrenia/ or Acute Stress/ or Acute Stress Disorder/ or Falls/ or Cerebrovascular Disorders/ or Critical Illness/ or Serious Mental Illness/	56975
9	((catastroph* or critica* or life-threatening or fatal* or emergen* or severe or detrimental or disast* or crisis* or crises* or unplanned or trauma* or sudden or unexpect* or premature or accident* or destruct* or cataclysm* or acute) adj2 (event* or outcome* or condition* or consequence* or disease* or failure* or fail* or accident* or clog* or ruptur* or pain* or toxicit* or infect* or complicat* or obstruct* or wound* or distress or dysphagia or depression* or depressed* or inflamm* or inflamed or stent* or bleed* or rebleed* or recurrent* or recur* or visit* or revisit* or admission* or admit* or readmit* or readmission* or reoperat* or experienc* or report* or inciden* or syndrome* or symptom*)).mp.	198639
10	(sudden* or premature or earl* or unexpect*) adj2 (die or died or death* or decease* or fatal*).mp.	7336
11	(suicid* or self-harm* or self-abus* or self-injur* or self-mutilat* or self-damag* or self-inflict*).mp.	101226
12	OR [7-11]	316326
Combining all three concepts		
13	3 AND 6	7
14	3 AND 12	172
15	13 OR 14	177
16	Limit 15 to (English language and yr='2015 – Current')	72

Supplementary S3 Data dictionary for two data extraction forms

Variable	Description	Possible values
**Table 1**		
Source	First author's last name, publication year	Astrup 2015
Country	Study country (if an international cohort was used, report all countries involved)	USA
Study design	Setting (single- or multi-center, provincial, national, international) and the type of the study sample (retrospective or prospective cohort or cross-sectional)	Single-center prospective cohort
Study cohort	A brief description of participants and the sample size	Adults undergoing radiation (n=133)
Age	Report the mean age ± SD (if available). Pool the overall mean if group-specific means were reported. Otherwise report the median (IQR) or the range (min-max) or the proportion of the most elderly group.	60 ± 11,<br>Median 64.1, range 54.4-73.5,<br>55 or older, 70.3%
Male	Report the proportion of study participants that were male. Pool the overall proportion if group-specific proportions of male were reported	70.0%
Cancer site	Report all subsites of HNC evaluated in a study. If no subsites were specified, report 'unspecified'	Oral cavity
Advanced disease	Report the proportion of the study participants that had pathologic or clinical stage III or IV at time of the initial assessment. Otherwise report 'Unknown'	61.0%
Treatment intent	Report the types of treatment intent among participants	Curative, Palliative, Both
**Table 2**		
Source	First author's last name, publication year	Astrup 2015
Phase of care	Report the phase of care when this catastrophic health event occurred in a study.	Postoperative, During curative treatment, Post-diagnosis
Outcome	Report the name of the outcome representing the catastrophic health event. For mortality, also report the time since last clinical contact if not 30 days. For other non-fatal outcomes, report the time since last clinical contact if <30 days	Unplanned readmission, Early death (6 months)
Incidence	Report the incidence of a catastrophic health event in the study participants. Pool the overall incidence if group-specific incidences were reported. Report separately if more than one catastrophic health events were reported.	In-hospital death 3.4%
Risk factors	Report all risk factors found through a multivariable regression analysis to be significantly associated with the incidence of a catastrophic health event using a 2-sided p-value<0.05 to indicate significance.	Older age, socially deprived, diabetes

Abbreviations: SD, standard deviation; IQR, interquartile range.

Supplementary S4 Process of using the Google NotebookLM to assist data extraction

First prompt: Hi NotebookLM, please study my Table 1 and hold on. Note my wordings, language, and tone for each piece of data I pulled out from the studies.

Source	Country	Study design	Study cohort	Age	Male	Cancer site	Cancer stage	Treatment intent
Wang 2021 [1]	China	Retrospective single-center cohort	Adults with HNC admitted for or have recently undergone surgery (n=6,576)	48.6±3.2	61.1%	N/A	I/II (37.4%) III/IV (43.6%)	Curative
Kam 2015 [2]	USA	Retrospective national cohort	Adults diagnosed with primary HNC (n=350,413)	>18	54%	All	8% distant	Mostly curative
Kallurkar 2019 [3]	USA	Retrospective single-center cohort	All adults treated with radiation with self-report pain (n=53)	61.2±1.7	73.6%	Oral cavity, pharynx, larynx	I/II (15.2%) III/IV (49.0%)	Curative
Wang 2022 [4]	Taiwan	Retrospective multi-center cohort	Adult HNC presenting to ED due to bleeding, hemorrhage, or carotid blowout followed up for rebleeding events (n=231)	56.7±10.9	93.1%	N/A	N/A	Mostly curative

Second prompt: Here is the first paper. I have also attached it as a PDF. Collect the same information from the attached PDF paper as shown in my table (Source, Country, Study design, Study cohort, Age, Male, Cancer site, Cancer stage, Treatment intent). Remember to keep it very simple and authentic to how I collected the information in my original table. [Insert paper]

Third prompt: Hi NotebookLM, please study my Table 2 and hold on. Note my wordings, language, and tone for each piece of data I pulled out from the studies.

Source	Phase of care	Outcomes and time window	Incidence	Risks factors
Wang 2021 [1]	Perioperative	Death (2-12d post-op)	7/6576 (0.11%)	Preop radiation, previous chemotherapy, hypertension, diabetes, advanced stage, postop infection
Kam 2015 [2]	All	Suicide (after diagnosis)	37.9/100,000 person-years	Male, advanced stage, age 60-79, hypopharynx or larynx cancer, radiation-only treatment
Wang 2022 [4]	All	Catastrophic (re)bleeding	112/231 (48.5%)	Laryngeal cancer, chemoradiation, second primary cancer

Source	Phase of care	Outcomes and time window	Incidence	Risks factors
		(after an index ED for bleeding)		
Kallurkar 2019 [3]	Post-radiation	Severe pain (>3mo after radiation completion)	13/53 (24.5%)	Pharynx cancer, squamous cell carcinoma, use of opioids

Fourth prompt: Here is the first paper. I have also attached it as a PDF. Collect the same information from the attached PDF paper as shown in my table (Source, Phase of care, Outcomes and tine window, Incidence, Risk factors). Remember to keep it very simple and authentic to how I collected the information in my original table. Risk factors are those with p-value<0.05 in multivariable regression analysis. [Insert paper]

References:
1. Wang, Y.; Wang, M.; Tang, Y.; Sun, B.; Wang, K.; Zhu, F. Perioperative Mortality of Head and Neck Cancers. *BMC Cancer* **2021**, *21*, 256, doi:10.1186/s12885-021-07998-z.
2. Kam, D.; Salib, A.; Gorgy, G.; Patel, T.D.; Carniol, E.T.; Eloy, J.A.; Baredes, S.; Park, R.C.W. Incidence of Suicide in Patients With Head and Neck Cancer. *JAMA Otolaryngol Head Neck Surg* **2015**, *141*, 1075, doi:10.1001/jamaoto.2015.2480.
3. Kallurkar, A.; Kulkarni, S.; Delfino, K.; Ferraro, D.; Rao, K. Characteristics of Chronic Pain among Head and Neck Cancer Patients Treated with Radiation Therapy: A Retrospective Study. *Pain Research and Management* **2019**, *2019*, 1–8, doi:10.1155/2019/9675654.
4. Wang, C.-K.; Ho, C.-F.; Niu, K.-Y.; Wu, C.-C.; Chang, Y.-C.; Hsiao, C.-H.; Yen, C.-C. Risk Factors for Rebleeding and Long-Term Outcomes in Patients with Head and Neck Cancer Bleeding: A Multicenter Study. *BMC Cancer* **2022**, *22*, 841, doi:10.1186/s12885-022-09945-y.

Table S1 Mapping risk factors for sudden or premature deaths

Source	Phase of care	Outcome	Male	Older age	SES	Comorbidity	HNC site	Advanced stage	Treatment
Kouka 2022	Post-diagnosis	Death (30-180d)	*	*			*	*	
Tu 2023	Post-diagnosis	Suicide		*	*	*	*	*	
Talani 2024b	Post-diagnosis	Death (6m)	*	*		*	*	*	
Bollig 2022~~1~~	Post-op	Death		*				*	
Wong 2025	Post-op	Death				*			
Mirza 2019	Post-op	In-hospital death			*	*	*	*	Invasive, emergent surg
Haapio 2016	Post-op	CVD death		*		*			
Zeng 2022	Post-op	In-hospital death				*			
Chaudhary 2017	Post-op	Death				*			Long LOS, unplanned
L'Esperance 2018	Post-op	Death (90d)		*		*			Large extent resection
Zhan 2016	Post-op	Death							Unplanned
Raikundalia 2016	Post-op	Death				*			
Frauenfelder 2021	Post-op	Deaths at ICU or hospital		*		*			Ventilation
Nieman 2018	Post-op	In-hospital death		*	*	*			Emergent, major surg, low-volume hospital
Oliver 2022	Post-op	Early death		*		*		*	Low-volume hospital
Shaikh 2023	Post-op	Death							Long OR time, 2-team
Lin 2020	Post-treatment	Death (90d)		*		*			
Talani 2024	Post-treatment	Early death				*			
Bazina 2025	Curative treatment	Early death (6m after completion)		*				*	
Voora 2022	Post-discharge	Death							Non-teaching hospitals
Yen 2022	Post-ED	Death							Inotropic support
Wang 2022	Post-ED	Death						*	
Total No. of studies			2	11	3	14	4	8	10

Unless otherwise stated outcomes were measured 30 days after the last clinical contact (e.g., completion of surgery).

Abbreviations: Post-op, postoperative; SES, socioeconomic status; HNC, head and neck cancer; surg, surgery; CVD, cardiovascular disease; LOS, length of stay; ICU, intensive care unit; OR, operation; d, day; m, month.

Table S2 Mapping risk factors for treatment-related complications

Source	Phase of care	Outcome	Sex	Older age	SES	Comorbidity	HNC site	Treatment
Wong 2025	Post-op	Hemorrhage				*	*	
Li 2019	Post-op	Hematoma, jugular rupture, carotid blowout						Surgical site infection
Wang 2022	After ED visit	Rebleeding				*	*	Chemoradiation
Crosetti 2016	Post-op	Complications						Invasive, long OR time
Mirza 2019	Post-op	Complications		*		*		Emergent, invasive surg
Luo 2025	Post-op	Complications						High dose steroid postoperative
Luo 2024	Post-op	Complications				*		
Haapio 2016	Post-op	Major cardiac and cerebrovascular events		*		*		
Zeng 20232	Post-op	Major complications				*		
L'Esperance 2018	Post-op	Serious complications				*		Long OR time
Sylvester 2017	Post-op	Complications				*		Partial or total laryngectomy
Raikundalia 2016	Post-op	Complications				*		
Lee 2015b	Post-op	Surgical site infections				*		
Koenen 2024	Post-op	Grade III-IV complications				*		Reconstruction
Nieman 2018	Post-op	Complications			*	*		Major/flap surg, urgent admission
Salati 2023	Post-op	Complications (fistula)				*		Flap reconstruction
Shaikh 2023	Post-op	Complications						Long OR time, 2-team
Sasaki 2015	During chemotherapy	Severe infections, febrile neutropenia				*		TPF regimen
Yao 2021	During chemo(radio)therapy	Leukopenia, bone marrow toxicity, GI injury	Female			*		IMRT
Total No. of studies			1	2	1	15	2	13

Unless otherwise stated outcomes were measured 30-days after the last clinical contact (e.g., completion of surgery).

Abbreviations: SES, socioeconomic status; OR, operation; post-op, postoperative; GI gastrointestinal; TPF, Taxotere-Platinol-Fluorouracil; IMRT, intensity-modulated radiation therapy.

Table S3 Mapping risk factors for unplanned acute care encounters

Source	Postoperative outcome	Male	Older age	SES	Comorbidity	HNC site	Advanced stage	Surgical
Wong 2025	Readmission, reoperation				*			
Foley 2023	Readmission			*	*		*	
Chiesa-Estomba 2022	Readmission, ED visit				*			Long LOS
Chaudhary 2017	Readmission		*	*	*		*	Long LOS
Zhao 2018	Reoperation						*	Vent, BT, wounds
Sangal 2018	Reoperation			*	*		*	Vent, long OR time, wounds
Ferrandino 2018	Readmission			*	*			Long LOS, vent
Osborn 2018	Readmission						*	Wounds
Helman 2017	Readmission or reoperation				*			Wounds
Carniol 2017	Readmission				*		*	BT, wounds
Zhan 2016	Readmission			*	*		*	
Badr 2019	Readmission, ED visit	*			*			
Chiou 2024	Readmission, ED revisit (≤3d)		*		*	*	*	Bilateral neck
Choi 2018	Reoperation						*	Long OR time
Frenkel 2018	Readmission, ED visit							Late g-tube
Garg 2017	Readmission				*		*	BT, wounds
Ghiam 2018	Readmission				*	*	*	Invasive, flap surg
Goel 20198	Non-elective readmission			*	*		*	Long LOS, admitted via ED, invasive surg
Madrigal 2023	Readmission				*			
Sindhar 2019	Readmission			*			*	
Voora 2022	Readmission		*		*			
Noel 2021	ED visits or hospital admissions after an outpatient ESAS assessment (14d)	*		*	*			Recent chemoradiation with or without surgery; high ESAS scores
Total no. of studies		2	3	8	17	2	13	15

Unless otherwise stated outcomes were measured 30 days after a head and neck cancer surgery.

Abbreviations: SES, socioeconomic status; HNC, head and neck cancer; ED, emergency department; OR, operation; LOS, length of stay; Vent, (mechanical) ventilation; BT, blood transfusion; g-tube, gastrostomy tube; d, day; ESAS, Edmonton Symptom Assessment System.

Table S4 Mapping risk factors for patient-reported severe symptoms

Source	Phase of care	Outcome	Age	SES	Comorbidity	HNC site	Advanced stage	Treatment
Chung 2025	Post-diagnosis	Suicidal ideation	Younger	*	*			Emergent admission, longer LOS
Lee 2015	Post-RT	Moderate or severe dry mouth			*			High dose of radiation
Astrup 2015	During RT	Moderate-to-severe pain		*	*			No surg before RT
Schaller 2021	During RT	Opioid for severe pain				*	*	Concurrent chemo
Klingelhoffer 2019	Post-op	Impaired swallow (7d)					*	Flap reconstruction
Van Abel 2022	Post-op	Severe pain (≤2d)	Younger				*	
Total no. of studies			2	2	3	1	3	5

Unless otherwise stated outcomes were measured 30 days after the last clinical contact.

Abbreviations: SES, socioeconomic status; HNC, head and neck cancer; post-op, postoperative; RT, radiotherapy; OR, operation; LOS, length of stay; surg, surgery; chemo, chemotherapy; d, day.
